# Superficial venous arterialization with the great saphenous vein *in situ*: a single-center experience

**DOI:** 10.1590/1677-5449.202300772

**Published:** 2024-03-25

**Authors:** Karlene Thayane Barros da Silva, Marcos Antonio Bonacorso Manhanelli, José Francisco Moron Morad, Fábio Linardi, José Augusto Costa

**Affiliations:** 1 Pontifícia Universidade Católica de São Paulo - PUC-SP, Faculdade de Ciências Médicas e da Saúde, São Paulo, SP, Brasil.; 2 Conjunto Hospitalar de Sorocaba - CHS, Sorocaba, SP, Brasil.

**Keywords:** critical ischemia, limb preservation, limb salvation, arterialization of the venous arch of the foot

## Abstract

**Background:**

Arterialization of the dorsal venous arch of the foot is a technique indicated in cases of critical lower limb ischemia that do not have a distal bed that is adequate to enable conventional treatment such as revascularization, angioplasty, or clinical treatment.

**Objectives:**

The purpose of this study is to present the result of arterialization of the venous arch of the foot in 16 patients who underwent treatment with this technique.

**Methods:**

This is a cross-sectional retrospective descriptive analytical study based on a review of the medical records of 16 patients who underwent arterialization of the dorsal venous arch of the foot for limb salvage from January 2016 to January 2021.

**Results:**

Four (25%) of the 16 patients who underwent arterialization of the venous arch of the foot underwent a major amputation during the same hospital stay and one patient (6.25%) had a major amputation within 6 months. The other 11 patients (68.75%) had their limbs preserved, with 10 undergoing minor amputations (toes and forefoot) and one patient having no additional procedures.

**Conclusions:**

We conclude that the technique of arterialization of the dorsal venous arch of the foot should be considered in selected cases. It is a valid alternative for limb salvage when conventional treatment is impossible.

## INTRODUCTION

The first attempts at arterialization of the venous arch of the foot were described in 1970 by Lengua who was attempting to supply arterial blood in the retrograde direction through the venous system with the intention of improving peripheral perfusion and support ulcer healing.^1-8^ The technique provokes reverse flow through the capillaries, improving tissue perfusion, increasing flow through preexisting collaterals and stimulating angiogenesis.^2,3,5,6,8,9^ It is indicated in cases of critical limb threatening ischemia^4,5^ with no adequate distal bed that would enable conventional treatment targeting limb salvage.^1,3,4^ It is an option when other alternatives are not possible or have already been attempted.^2,5,10,11^

The success of this technique depends on its indication in selected cases with appropriate preoperative preparation and detailed arterial and venous studies, such as arteriography and arterial and venous Doppler.^1,5^ Graft patency should be verified in the immediate postoperative period, in addition to checking limb temperature and reduction of pain.^4,9^

This is a technique for use in exceptional circumstances with the objective of avoiding limb loss, since approximately 50% of these patients die within 1 year and a large proportion of them are considered unsuitable for rehabilitation because of existing comorbidities. The biopsychosocial disorders inherent to major amputations compound this.^2,4,8,12-14^

The objective of this study was to present the results of arterialization of the venous arch of the foot performed in 16 patients with critical ischemia at imminent risk of limb loss.^1,5,9,15^

## METHODS

This is a cross-sectional retrospective descriptive analytical study based on review of the medical records of patients who underwent arterialization of the dorsal venous arch of the foot for limb salvage, from January 2016 to January 2021, at a single center. The study was submitted to and approved by the Ethics Committee (Ethics Appraisal Submission Certificate No. 65876022.1.0000.5373, consolidated opinion No. 5.812.022).

The initial sample comprised patients who had the term “arterialization” in the descriptions of their surgery. These records were identified and reviewed and patients were selected who had undergone arterialization of the venous arch of the foot. Medical records were excluded if the descriptions of surgery did not confirm that arterialization had been performed, including medical records in which the term was mentioned in progress notes or in other documents as a treatment option.

The review identified 17 medical records, one of which was excluded from the sample because of incomplete data.

During the period from January 2016 to January 2021, 16 patients admitted to the Conjunto Hospitalar de Sorocaba (CHS) underwent arterialization of the venous arch of the foot and were maintained in outpatient follow-up for 1 year. They had Rutherford classifications 4, 5, or 6 and had angiographic studies demonstrating absence of an adequate distal arterial bed for conventional treatment.

**Figure 1:**
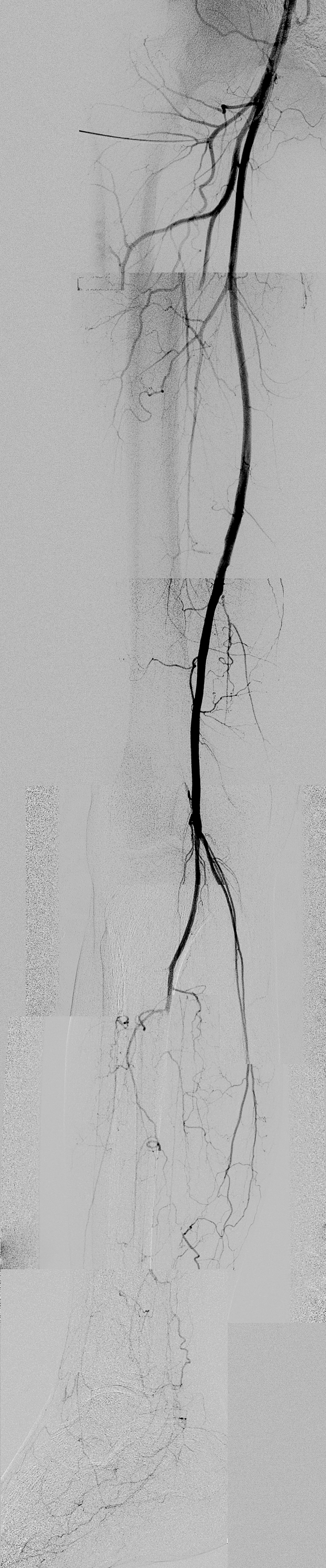
Arteriography demonstrating absence of distal bed.

All of these patients underwent arteriography (Figure 1) and duplex arterial mapping to assess the distal bed and to determine the best choice of donor artery.

Duplex venous mapping was used to assess the superficial veins, in particular the characteristics of the saphenous vein and the deep system, with the objective of testing its patency for provision of runoff for the extra flow that would result from creation of an arteriovenous fistula.

**Figure 2:**
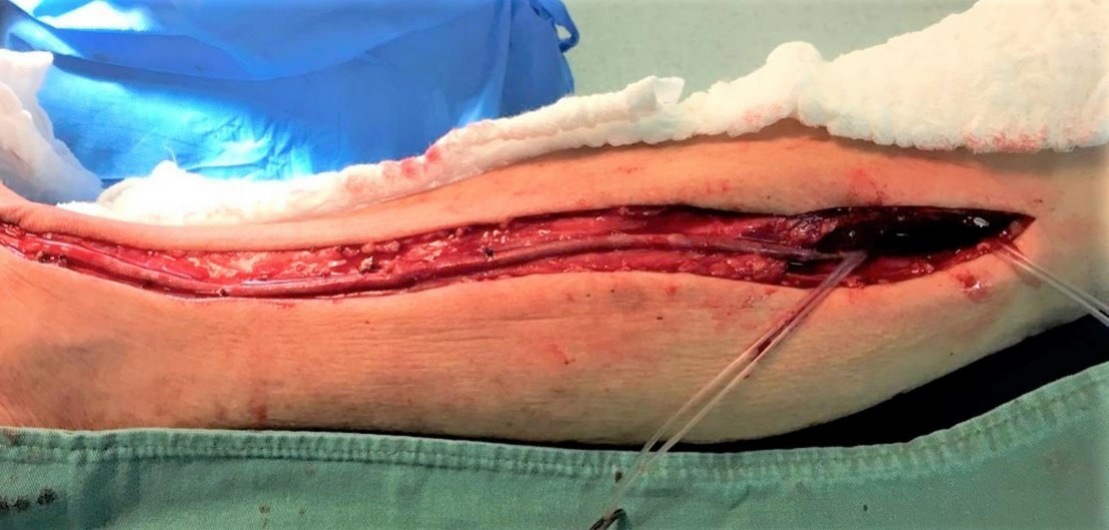
Exposure of the great saphenous vein and ligature of tributaries.

With relation to the technical aspects of the surgery, the first step was dissection and exposure of the great saphenous vein in its bed and ligature of all its tributaries (Figure 2).

**Figure 3:**
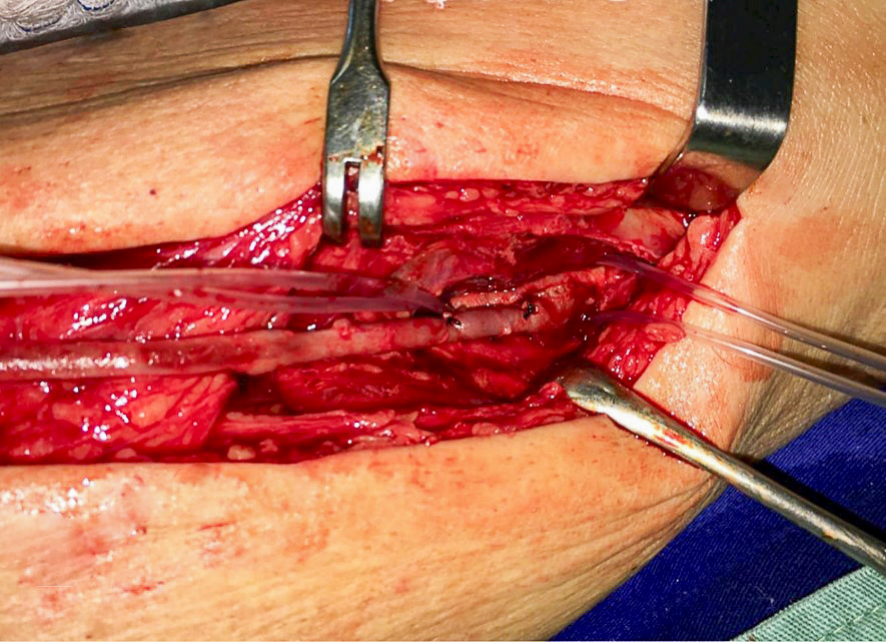
End-to-side anastomosis of the great saphenous vein with the infrapatellar popliteal artery.

A Mills valvulotome (Otemab R) was introduced via the most distal tributary possible that had adequate caliber. The most often used was the medial plantar vein, when possible, or a venotomy was performed more proximal to the malleolus. The proximal anastomosis was performed as distal as possible to the femoral artery, or to the popliteal artery, using 6-0 prolene suture (Figure 3).

During the immediate postoperative period, presence of pulse along the course of the saphenous vein was checked and flow was verified using a portable Doppler machine. Other clinical signs were also assessed, such as increased limb temperature, limb color, and perfusion time.^4,9^

Variables were input to a database constructed in Microsoft Office Excel® 2010.

## RESULTS

Male patients accounted for 75% of the sample (n=12). Ages ranged from 33 to 79 years (mean of 61.7 years). The distribution of patients by age brackets is shown in Table 1.

**Table 1:** Distribution of patients by age groups.

**Age (years)**	**N**	**%**
30 to 39	2	12.50
40 to 49	1	6.25
50 to 59	2	12.50
60 to 69	7	43.75
70 to 79	4	25.00

The most prevalent clinical condition was peripheral arterial disease (PAD), in 11 patients, followed by acute arterial occlusion (AAO), in 3 patients, and thromboangiitis obliterans (TAO), in 2 patients. All of the patients with PAD had trophic lesions, 10 with well-delimited ulcers (Rutherford 5) and 1 with major tissue lesions (Rutherford 6).

All of the patients with TAO had well-delimited trophic lesions, located on the great toe or lesser toes (Rutherford 5). Clinical presentation in the patients with AAO was restricted to pain at rest and hypothermia of the limb (Rutherford 2A) (Table 2).

**Table 2:** Distribution of patients by etiology and Rutherford classification.

**Disease**	**N**	**%**	**Rutherford**
Peripheral arterial disease	11	68.75	10 R5
			1 R6
Acute arterial occlusion	3	18.75	2A
Thromboangiitis obliterans	2	12.50	R5

The most common comorbidities were systemic arterial hypertension, diabetes mellitus, heart disease, stroke, and chronic renal failure (Table 3). Smoking was the most common risk factor, present in 68.7% of the patients.

**Table 3:** Distribution of patients by comorbidities.

**Comorbidities**	**N**	**%**
Systemic arterial hypertension	10	62.50
Diabetes mellitus	9	56.25
Heart disease	3	18.75
Stroke	2	12.50
Chronic renal failure	1	6.25

The time elapsed before emergence of ulcers ranged from 7 days to 6 months, with a mean of 82.5 days.

Four patients progressed to graft failure, and remained in intense pain with pallor, coldness, absence of pulse along the course of the saphenous vein, and no flow detectable with the portable Doppler machine. All four underwent major amputation (transfemoral) during the same admission.

Three of the four patients who underwent major amputation during the same admission had PAD, one with major tissue loss (Rutherford 6) and two with focal trophic lesions (Rutherford 5), and the fourth patient had AAO (Rutherford 2A).

The other patients in the sample had good postoperative progress, with improvement of pain and limb color and pulses detectable along the course of the saphenous vein.

During the same admission, 11 patients underwent minor amputations, (of toes or at the level of the foot) and were discharged exhibiting good postoperative progress, while one patient was discharged without needing any additional procedures.

During outpatient follow-up, one patient with PAD and focal ulceration (Rutherford 5) exhibited deteriorating ischemia due to graft failure and underwent a major amputation (transfemoral) after 6 months of follow-up.

Table 4 presents patient distribution by etiology, Rutherford classification, and additional procedures.

**Table 4:** Distribution of patients by etiology, Rutherford classification, and additional procedures.

**Total n**	**Etiology**	**Rutherford classification**	**Major AMP**	**Minor AMP**	**No procedures**
**1**	PAD	R6	1	0	0
**10**	PAD	R5	3	7	0
**3**	AAO	2 A	1	1	0
**2**	TAO	R5	0	1	1

AMP = amputation; PAD = peripheral arterial disease; AAO = acute arterial occlusion; TAO = thromboangiitis obliterans.

Eleven patients were still free from recurrence of signs and symptoms of ischemia at 1-year follow-up.

## DISCUSSION

Conceptually, arterialization constitutes using the venous bed in a disease-free area as an alternative route to perfuse tissues with arterial blood, thereby averting major amputations.^1,10^ Approximately 20% of patients with critical lower limb ischemia do not have a distal bed adequate for conventional arterial reconstruction.^4,5,16^

Successful use of the technique is dependent on indicating it for selected cases in which the distal bed is compromised, making it impossible to achieve sufficient run-off via a conventional bypass.^1^

As such, arterialization is a way of attempting to avoid major amputations in patients with critical limb-threatening ischemia who are not suited for conventional revascularization.^17^ We consider that use of the technique is successful when ulcers heal or only a minor amputation is performed.^18,19^

The small and heterogeneous sample and the lack of supplementary tests from postoperative follow-up are limitations of this study.

This technique should be considered in the following situations:

- Absence of surgically accessible distal arterial bed (whether by conventional or endovascular surgery);
- Failed clinical treatment;
- Failed surgical revascularization (conventional or endovascular); or
- When the only other treatment option is limb amputation.

## CONCLUSIONS

The results presented here, with 68.75% of limbs salvaged, support the conclusion that this technique should be considered in selected cases with the objectives cited above.
