# Supplementary material for: Superficial venous arterialization with the great saphenous vein in situ: a single-center experience
Source: J Vasc Bras. 2024 Mar 25;23:e20230077. doi: 10.1590/1677-5449.202300772 (PMC10984586; doi:10.1590/1677-5449.202300772)
Supplement: Translation

# Translation: Arterialização venosa superficial com veia safena magna *in situ*: a experiência de um único centro

**DOI:** 10.1590/1677-5449.202300771

**Authors:** Karlene Thayane Barros da Silva, Marcos Antonio Bonacorso Manhanelli, José Francisco Moron Morad, Fábio Linardi, José Augusto Costa

**Affiliations:** 1 Pontifícia Universidade Católica de São Paulo - PUC-SP, Faculdade de Ciências Médicas e da Saúde, São Paulo, SP, Brasil.; 2 Conjunto Hospitalar de Sorocaba - CHS, Sorocaba, SP, Brasil.

**Keywords:** isquemia crítica, preservação do membro, salvamento de membro, arterialização do arco venoso do pé

## Abstract

**Contexto:**

A arterialização do arco venoso dorsal do pé é uma técnica indicada em casos de isquemia crítica de membros inferiores sem leito distal adequado que possibilite tratamento convencional, como revascularização, angioplastia ou tratamento clínico.

**Objetivos:**

O propósito do trabalho foi apresentar o resultado da arterialização do arco venoso do pé em 16 pacientes submetidos a essa técnica.

**Métodos:**

Tratou-se de um estudo analítico descritivo retrospectivo transversal, baseado na revisão de prontuários de 16 pacientes submetidos à arterialização do arco venoso dorsal do pé para salvamento de membro, entre janeiro de 2016 a janeiro de 2021.

**Resultados:**

Dos 16 pacientes submetidos à arterialização do arco venoso do pé, 25% (4) evoluíram para amputação maior durante a mesma internação, e 6,25% (1) pacientes evoluíram para amputação maior após 6 meses. Os demais pacientes (68,75%, 11) tiveram seus membros preservados, sendo que 10 foram submetidos a amputações menores (pododáctilos e antepé), e 1 paciente não necessitou de procedimento adicional.

**Conclusões:**

A técnica de arterialização do arco venoso dorsal do pé deve ser considerada em casos selecionados. Trata-se de uma alternativa válida para a preservação do membro na impossibilidade de tratamento convencional.

## INTRODUÇÃO

As primeiras tentativas de arterialização do arco venoso do pé foram descritas em 1970 por Lengua, ao tentar levar sangue arterial através do sistema venoso por via retrógrada, com o intuito de melhorar a perfusão periférica e promover a cicatrização de lesões^1-8^ . Essa técnica promove a reversão do fluxo nos capilares, melhorando a nutrição tecidual, aumentando o fluxo nas colaterais existentes e estimulando a angiogênese^2,3,5,6,8,9^ . Ela está indicada em casos de isquemia crítica ameaçadora de membros^4,5^ sem leito distal adequado que possibilite tratamento convencional com a finalidade de salvamento do membro^1,3,4^ . Torna-se uma alternativa quando as opções disponíveis não são possíveis ou já foram tentadas^2,5,10,11^ .

O sucesso dessa técnica depende da indicação em casos selecionados com preparo pré-operatório adequado e com estudo arterial e venoso detalhado, como arteriografia e Doppler arterial e venoso^1,5^ . No pós-operatório imediato, deve-se observar a patência do enxerto, a temperatura do membro e a redução da dor^4,9^ .

Trata-se de uma técnica de exceção com a finalidade de evitar a perda de membros, pois aproximadamente 50% desses pacientes evoluem a óbito em até 1 ano e uma grande parcela deles é considerada imprópria para reabilitação devido às comorbidades existentes. Acrescenta-se a isso os transtornos biopsicossociais inerentes às amputações maiores^2,4,8,12-14^ .

O objetivo deste trabalho foi apresentar os resultados obtidos com a arterialização do arco venoso do pé realizada em 16 pacientes com isquemia crítica com risco iminente de perda de membro^1,5,9,15^ .

## MÉTODOS

Tratou-se de um estudo analítico descritivo retrospectivo transversal, baseado na revisão de prontuários de pacientes submetidos à arterialização do arco venoso dorsal do pé para salvamento de membro, no período entre janeiro de 2016 a janeiro de 2021, em um único centro. O estudo foi submetido e aprovado pelo Comitê de Ética, com Certificado de Apresentação de Apreciação Ética n.º 65876022.1.0000.5373 e parecer consubstanciado n.º 5.812.022.

A amostra foi constituída por pacientes que possuíam o termo “arterialização” em suas descrições operatórias. Esses prontuários foram identificados e revisados, sendo selecionados dessa amostra inicial os pacientes que foram submetidos à arterialização do arco venoso do pé. Foram excluídos prontuários em que as descrições cirúrgicas não permitiam confirmar a realização da arterialização, sendo prontuários em que o termo foi citado em evoluções ou em outros documentos como alternativa ao tratamento.

Foram identificados 17 prontuários, sendo que um foi excluído da amostra devido ao preenchimento incompleto dos dados.

No período entre janeiro de 2016 e janeiro de 2021, 16 pacientes internados no Conjunto Hospitalar de Sorocaba (CHS) foram submetidos à arterialização do arco venoso do pé e acompanhados ambulatorialmente por 1 ano. Eles tinham classificação 4, 5 e 6 de Rutherford, com estudos angiográficos demonstrando ausência de leito distal arterial adequado para tratamento convencional.

Todos os pacientes foram submetidos à arteriografia (Figura 1) e ao mapeamento dúplex arterial com a finalidade de avaliar o leito distal, assim como verificar a melhor opção de artéria doadora.

**Figura 1:** Arteriografia demonstrando ausência de leito distal.

O mapeamento dúplex venoso avaliou as veias superficiais, em especial as características da veia safena e o sistema profundo, com a finalidade de verificar sua perviedade para dar vazão ao hiperfluxo ocasionado pela criação de uma fístula arteriovenosa.

Em relação aos aspectos técnicos da cirurgia, inicialmente foram realizadas a dissecção e a exposição da veia safena magna em seu leito e a ligadura de todas as tributárias (Figura 2).

**Figura 2:** Exposição da veia safena magna e ligadura das tributárias.

Foi introduzido um valvulótomo de Mills (Otemab R) através da tributária mais distal possível e com calibre adequado, sendo a veia plantar medial a mais utilizada quando possível ou por venotomia mais próxima do maléolo. A anastomose proximal foi realizada na artéria femoral mais distal possível ou na artéria poplítea, com fio prolene 6-0 (Figura 3).

**Figura 3:** Anastomose terminolateral da veia safena magna com artéria poplítea infrapatelar.

No pós-operatório imediato, foram avaliadas a presença de pulsatilidade no trajeto da veia veia safena e a presença de fluxo ao Doppler portátil. Outros sinais clínicos foram avaliados, como o aumento da temperatura do membro, a sua coloração e o tempo de perfusão^4,9^ .

As variáveis foram apuradas em banco de dados elaborado no *software* Microsoft Office Excel® 2010.

## RESULTADOS

Os pacientes do sexo masculino representaram 75% da amostra (12). A idade variou entre 33 e 79 anos (média de 61,7 anos). A distribuição dos pacientes de acordo com o intervalo de idade está apresentada na Tabela 1.

**Tabela 1:** Distribuição dos pacientes de acordo com faixa etária.

**Idade (anos)**	**N**	**%**
30 a 39	2	12,50
40 a 49	1	6,25
50 a 59	2	12,50
60 a 69	7	43,75
70 a 79	4	25,00

A condição clínica mais redundante foi a doença arterial periférica (DAP) em 11 pacientes, seguida de oclusão arterial aguda (OAA) em 3 pacientes e tromboangeíte obliterante (TAO) em 2 pacientes. Entre os pacientes com DAP, todos apresentavam lesão trófica, sendo 10 com lesão bem delimitada (Rutherford 5) e 1 com lesão extensa (Rutherford 6).

Todos os pacientes com TAO apresentavam lesão trófica bem delimitada, localizadas em hálux ou em pododáctilo (Rutherford 5). Nos pacientes com OAA, a clínica era somente de dor em repouso e hipotermia do membro (Rutherford 2A) (Tabela 2).

**Tabela 2:** Distribuição dos pacientes de acordo com a etiologia e a classificação de Rutherford.

**Doença**	**N**	**%**	**Rutherford**
Doença arterial periférica	11	68,75	10 R5
			1 R6
Oclusão arterial aguda	3	18,75	2A
Tromboangeíte obliterante	2	12,50	R5

As comorbidades mais frequentes foram hipertensão arterial sistêmica, diabetes melito, cardiopatia, acidente vascular cerebral e insuficiência renal crônica (Tabela 3). O tabagismo foi fator de risco presente em 68,7% dos pacientes.

**Tabela 3:** Distribuição dos pacientes de acordo com as comorbidades.

**Comorbidades**	**N**	**%**
Hipertensão arterial sistêmica	10	62,50
Diabetes melito	9	56,25
Cardiopatia	3	18,75
Acidente vascular cerebral	2	12,50
Insuficiência renal crônica	1	6,25

O tempo para o surgimento dessas lesões variou de 7 dias a 6 meses, com média de 82,5 dias.

Quatro pacientes evoluíram com falência do enxerto, permanecendo com dor intensa, palidez, frialdade, ausência de pulso em trajeto da veia safena e ausência de fluxo ao Doppler portátil. Todos foram submetidos a amputação maior (transfemoral) durante a mesma internação.

Entre os quatro pacientes submetidos a amputação maior durante a mesma internação, três eram portadores de DAP, sendo um com lesão extensa (Rutherford 6) e dois com lesão trófica limitada (Rutherford 5), e um paciente era portador de OAA (Rutherford 2A).

Os demais pacientes apresentaram boa evolução pós-operatória, com melhora da dor e da coloração e presença de pulso em trajeto da veia safena.

Durante a mesma internação, 11 pacientes foram submetidos a amputações menores (pododáctilos ou ao nível do pé) e receberam alta com boa evolução

pós-operatória, e um paciente recebeu alta sem necessidade de nenhum procedimento complementar.

Em seguimento ambulatorial, um paciente portador de DAP com lesão limitada (Rutherford 5) apresentou piora da isquemia por falência do enxerto e foi submetido a amputação maior (transfemoral) após 6 meses de acompanhamento.

A distribuição dos pacientes de acordo com a etiologia, a classificação de Rutherford e os procedimentos adicionais está representada na Tabela 4.

**Tabela 4:** Distribuição dos pacientes de acordo com a etiologia, a classificação de Rutherford e os procedimentos adicionais.

**N total**	**Etiologia**	**Classificação de Rutherford**	**AMP maior**	**AMP menor**	**Livre de procedimento**
**1**	DAP	R6	1	0	0
**10**	DAP	R5	3	7	0
**3**	OAA	2 A	1	1	0
**2**	TAO	R5	0	1	1

AMP = amputação; DAP = doença arterial periférica; OAA = oclusão arterial aguda; TAO = tromboangeíte obliterante.

Onze pacientes evoluíram sem recorrência de sinais e sintomas de isquemia em 1 ano de seguimento.

## DISCUSSÃO

Conceitualmente, a arterialização consiste em utilizar o leito venoso em local livre de doença como via alternativa para perfundir os tecidos com sangue arterial e, assim, evitar grandes amputações^1,10^ . Aproximadamente 20% dos pacientes com isquemia crítica de membro inferior não possuem leito distal adequado para reconstruções arteriais convencionais^4,5,16^ .

O sucesso dessa técnica depende de indicação em casos selecionados, quando ocorre comprometimento do leito distal que impossibilite o deságue adequado para uma derivação convencional^1^ .

Dessa forma, a arterialização consiste em uma forma de tentar evitar amputações maiores em pacientes com isquemia crítica ameaçadora de membros, sem possibilidade de revascularização convencional^17^ . Consideramos sucesso no uso dessa técnica quando ocorre cicatrização de feridas ou quando é realizada uma amputação menor^18,19^ .

A amostra pequena e heterogênea e a ausência de exames complementares no seguimento pós-operatório compreendem as limitações deste estudo.

A técnica deve ser considerada nas determinadas situações:

- Ausência de leito arterial distal cirurgicamente acessível (seja por via cirúrgica convencional ou por via endovascular);
- Falha do tratamento clínico;
- Falha da revascularização cirúrgica (convencional ou endovascular); ou
- Quando a única opção de tratamento é a amputação do membro.

## CONCLUSÃO

Os resultados apresentados, com 68,75% de salvamento de membros, permitem concluir que a técnica deve ser considerada em casos selecionados com os objetivos supracitados.
